# Prolonged Organ Extraction Time Negatively Impacts Kidney Transplantation Outcome

**DOI:** 10.3389/ti.2021.10186

**Published:** 2022-02-09

**Authors:** Hanno Maassen, Henri G. D. Leuvenink, Harry van Goor, Jan-Stephan F. Sanders, Robert A. Pol, Cyril Moers, H. Sijbrand Hofker

**Affiliations:** ^1^ Department of Surgery, University Medical Center Groningen, University of Groningen, Groningen, Netherlands; ^2^ Department of Pathology and Medical Biology, University Medical Center Groningen, University of Groningen, Groningen, Netherlands; ^3^ Department of Nephrology, University Medical Center Groningen, University of Groningen, Groningen, Netherlands

**Keywords:** extraction, time, nephrectomy, kidney, transplantation outcome

## Abstract

**Main Problem:** Following cold aortic flush in a deceased organ donation procedure, kidneys never reach the intended 0–4°C and stay ischemic at around 20°C in the donor’s body until actual surgical retrieval. Therefore, organ extraction time could have a detrimental influence on kidney transplant outcome.

**Materials and Methods:** We analyzed the association between extraction time and kidney transplant outcome in multicenter data of 5,426 transplant procedures from the Dutch Organ Transplantation Registry (NOTR) and 15,849 transplant procedures from the United Network for Organ Sharing (UNOS).

**Results:** Extraction time was grouped per 10-min increment. In the NOTR database, extraction time was independently associated with graft loss [HR 1.027 (1.004–1.050); *p* = 0.022] and with DGF [OR 1.043 (1.021–1.066); *p* < 0.005]. An extraction time >80 min was associated with a 27.4% higher hazard rate of graft failure [HR 1.274 (1.080–1.502); *p* = 0.004] and such kidneys had 43.8% higher odds of developing DGF [OR 1.438, (1.236–1.673); *p* < 0.005]. In the UNOS database, increasing extraction times in DCD donors were associated with DGF [OR 1.036 (1.016–1.055); *p* < 0.005]. An extraction time >30 min was associated with 14.5% higher odds of developing DGF [OR 1.145 (1.063–1.233); *p* < 0.005].

**Discussion:** Prolonged kidney extraction time negatively influenced graft survival in Dutch donors and increased DGF risk in all deceased donor recipients.

## Graphical Abstract

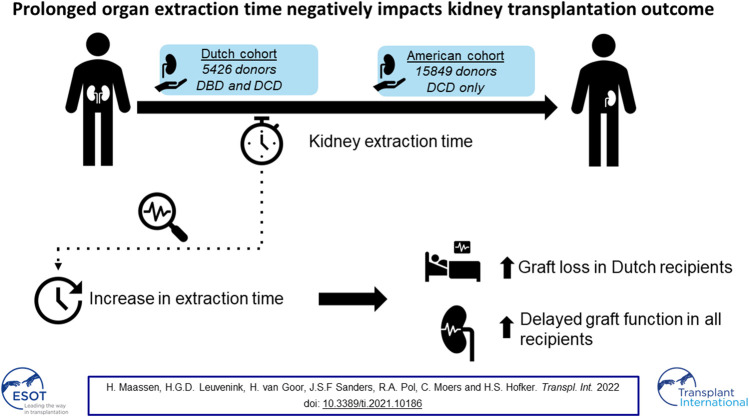

## Introduction

Kidney transplantation is the preferred treatment for end-stage chronic kidney disease (1). Although kidney transplant outcomes have improved over time and new preservation techniques show promising results, (2) further improvements may be possible. In deceased donor organ procurement, the extraction time is the time interval between the start of the cold flush with preservation solution through an aortic cannula and the actual extraction of the organ from the body. The aim is to reduce temperature and metabolism, and thus protect organs against ischemic injury. Unfortunately, despite the cold flush and topical cooling of the abdominal cavity with slushed ice, the temperature of the kidneys does not reach the intended 4°C required to fully minimize metabolism (3) and remains up to around 20°C right before the actual extraction (4). This is in line with liver procurement, where the organ does not reach the preferred 4°C during procurement surgery either (5, 6). In deceased liver donation, prolonged liver extraction time has been shown to impair liver transplant outcome (7). Prolonged kidney extraction time could also be detrimental to organ quality and kidney transplantation outcome. The effect of the extraction time on kidney graft function is a subject of debate. Data from a single organ procurement organization showed a higher risk of delayed graft function (DGF) with increasing extraction time (8). Another study found no association between extraction time and early graft failure, DGF or graft survival, but there was an association between extraction time and rate of recovery from DGF (9). Unfortunately, the relatively small number of kidney transplantations analyzed in these studies restricts generalization of findings. Heylen et al. analyzed the Eurotransplant database in a multicentre cohort study and found that prolonged extraction time was associated with graft loss after donation after circulatory death (DCD), but not after brain death donation (DBD) (10). The current study analyzed multicenter data of transplant procedures in the Netherlands and the United States, aiming to determine an association between kidney extraction time and post-transplantation kidney function, DGF, graft failure and possibly patient mortality.

## Methods and Materials

### Study Population

Data on all kidney transplantations performed between January 2002 and December 2016 were obtained from the Dutch Organ Transplant Registry (NOTR). Consent for the conduct of this retrospective database study was obtained from the Netherlands Transplantation Foundation data governance board, representing all Dutch transplant centers. Deceased donor, DBD and DCD, and recipient data were analyzed. Follow-up data up to May 2018 were available.

Data from the United Network for Organ Sharing (UNOS) were also used. Data submitted to the registry between February 2005 and March 2019 were analyzed. In the UNOS database extraction time was only available for DCD donors, hence no analyses could be performed on DBD donor kidneys from this database. Follow-up data up to June 2019 were available. Studies using the UNOS dataset are exempt from review by the Institutional Review Board.

**FIGURE 1:**
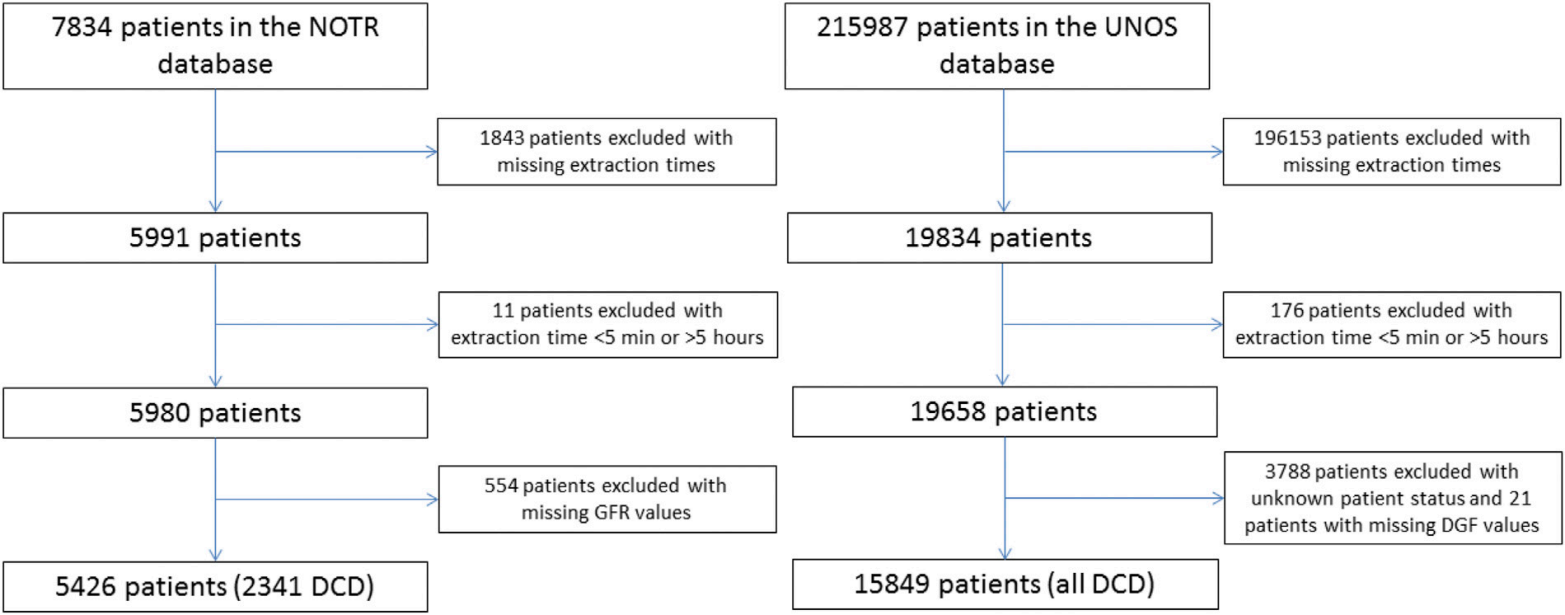
Flowchart exclusion of patients in both the NOTR database and the UNOS database.

Inclusion criteria were all deceased kidney transplantations with available extraction times. Exclusion criteria were extraction times under 5 min or over 5 h and missing outcome data (i.e., patient survival, graft failure and DGF, and in the NOTR database unknown renal function at 3 months post-transplantation) (Figure 1).

Extraction time of the kidney was calculated and defined as start of the cold aortic flush until end of nephrectomy, and times were grouped in 10-min increments. In addition to extraction time, warm ischemic time (DCD only), cold ischemic time, and anastomosis time were defined accordingly to the Eurotransplant manual (11). Post-transplantation estimated Glomerular Filtration Rate (eGFR) was calculated in the NOTR according to the MDRD formula: 186 × (creatinin/88.4)^−1.154^ × (age)^−0.203^ × (0.742 with female). In the UNOS database, necessary data was missing for calculation of the eGFR post-transplantation. Patient survival was defined as the time from transplantation until death. Graft survival was defined as the time from transplantation until failure of the graft, death-censored and it includes all causes of graft failure. DGF was defined as any dialysis requirement in the first week post transplantation.

### Kidney Donor Risk Index

The Kidney Donor Risk Index (KDRI) was calculated in the Dutch database using a standardized formula including age, height, weight, history of hypertension, history of diabetes, cause of death, serum creatinine and DCD status (12). Hepatitis C virus (HCV) status and ethnicity were not available in the database, therefore we assumed all patients were Caucasian and were not infected with HCV. HCV infection was stated to be 0.2% in other research conducted with Dutch transplant donors and recipients (13). First, KDRI_rao_ was calculated with the previously mentioned variables. Next, KDRI_median_ was calculated using the same scaling factor used in the UNOS database (1.250695754). The KDRI was already available in UNOS, so no further calculations were performed on those data.

### Statistical Analysis

Statistical analysis was performed on cases for which extraction time and outcome was available using multivariable Cox regression for patient survival and graft failure, logistic binary regression for DGF and graft rejection or multivariable linear regression for exploring factors influencing extraction time. Missing values for the variables history of hypertension and history of diabetes were defined as “not present.” Median values were imputed in the NOTR database for missing values in the variables “reported number of organs” (1,014 cases), warm ischemic time (631 cases), cold ischemic time (576 cases), second ischemic time (642 cases), HLA mismatches (29 cases), body mass index (BMI) (2 cases) and KDRI median (8 cases). Baseline characteristics are presented as median with range or number with percentage. Univariable variables were tested for normal distribution and comparisons between groups were performed accordingly. Pearson’s chi-square test was used for donor sex, donor hypertension, donor diabetes, recipient sex, DGF occurrence and previous kidney transplantations, Mann-Whitney U-test for donor age, donor BMI, cold ischemic time, extraction time, extraction time kidney-only donation, KDRI_median_, recipient age and HLA mismatches and log-rank test for 5 years graft survival. Performance of univariable analysis and determination of potential confounders were followed by a stepwise multivariable analysis. A *p*-value of ≤0.05 was assumed to be statistically significant. An interaction analysis was performed for DBD/DCD and extraction time in the NOTR database based on model 5 of Table 2, with the addition of DBD/DCD*extraction time for outcomes with a significant association with extraction time. Extraction time was dichotomized to perform a cut-off value analysis. After dividing the data binary, multiple analysis were performed to find the cut-off value. Multivariable cox regression was used for patient survival and graft failure, binary logistic regression was used for DGF. An increase in familywise error rate was controlled by Bonferroni correction. Leading to a *p*-value of 0.00625 to be regarded statistically significant at the cut-off analysis part of the manuscript. Statistical analysis was performed using SPSS Statistics version 23.

## Results

### Donor and Recipient Characteristics

Donor and recipient characteristics are shown in Table 1, displaying NOTR data with DBD and DCD donors, NOTR data with only DCD donors, and UNOS data (the extraction time was only available in DCD donors in the UNOS data). Notable differences between DBD and DCD donors from the Dutch database are a higher rate of males among DCD donors (DCD: 58.7% vs. DBC: 46.7%) and the occurrence of more DGF in DCD versus DBD kidney recipients (DCD: 52.7% vs. DBD: 19.4%). Median donor age was much higher in DCD donors from the NOTR database compared to UNOS [52, (1–78) vs. 39 (1–69), *p* < 0.005]. Of the 2341 DCD donors, 58.6% were male in NOTR compared to 66.5% in 15849 in the UNOS database (*p* < 0.005). Table 1 shows the number of reported organs in NOTR and the number of extracted organs in UNOS. The number of extracted organs per donor was not available in NOTR. Cold ischemic time was significantly shorter in NOTR [NOTR: 961 min (119–2797) vs. UNOS: 1080 min (0.6–5940), *p* < 0.005], with a significantly longer median kidney extraction time (NOTR: 59 min vs. UNOS: 38 min, *p* < 0.005). In the kidney-only donation, the median extraction time was also significantly longer in NOTR (NOTR: 53 min vs. UNOS: 33 min, *p* < 0.005). KDRI_median_ was significantly higher in NOTR, showing on average a better quality of donors from UNOS [NOTR: 1.099 (0.57–2.35) vs. UNOS: 0.9515 (0.56–2.49), *p* < 0.005].

**TABLE 1:** Donor and Recipient Characteristics of NOTR (Jan 2002–Dec 2016) and UNOS (Feb 2005–Mar 2019) databases.

Characteristics	NOTR all (5426)	NOTR DCD only (2341)	UNOS DCD only (15849)	DCD NOTR vs UNOS
Donor				
Age, years	52 (1–86)	52 (1–78)	39 (1–69)	*p* < 0.005
Sex				*p* < 0.005
Male	2814 (51.9%)	1374 (58.7%)	10542 (66.5%)	
Female	2612 (48.1%)	967 (41.3%)	5307 (33.5%)	
BMI	24.7 (9.8–55.6)	24.7 (12.5–55.6)	26.9 (8.91–69.2)	*p* < 0.005
Donor Type				
DBD	3085 (56.9%)			
DCD	2341 (43.1%)	2341 (100%)	15849 (100%)	
Cause of Death				
CVA	1406 (25.9%)	538 (23%)	2455 (15.5%)	
Trauma	1153 (21.2%)	609 (26%)		
Head trauma			5089 (32.1%)	
Anoxia			7543 (47.6%)	
Other	2867 (52.8%)	1194 (51%)	762 (4.8%)	
Hypertension				*p* < 0.005
Yes	1243 (22.9%)	448 (19.1%)	3778 (23.8%)	
No	4183 (77.1%)	1893 (80.9%)	12071 (76.2%)	
Diabetes				*p* < 0.005
Yes	269 (5%)	115 (4.9%)	903 (5.7%)	
No	5157 (95%)	2226 (95.1%)	14946 (94.3%)	
Reported number of organs (NOTR)*			Extracted no. organs (UNOS)*	
1	5 (0.1%)	5 (0.2%)	56 (0.4%)	
2	669 (12.3%)	600 (25.6%)	8657 (54.6%)	
3	705 (13%)	531 (22.7%	5672 (35.8%)	
4	1851 (34.1%)	528 (22.6%)	866 (5.5%)	
5	544 (10%)	197 (8.4%)	527 (3.3%)	
6	846 (15.6%)	458 (19.6%)	71 (0.4%)	
7	806 (14.9%)	22 (0.9%)		
Warm ischemic time (DCD only), min		17 (6–54)	17 (0–180)**	
Cold ischemic time, min	961 (60–2880)	961 (119–2797)	1080 (0.6–5940)	*p* < 0.005
Anastomosis time, min	33 (10–180)	33 (11–180)		
Extraction time, min	58 (5–300)	59 (5–293)	38 (5–259)	*p* < 0.005
Extraction time kidney-only donation, min	52 (5–293)	53 (5–293)	33 (6–165)	*p* < 0.005
KDRI_median_	1.0395 (0.51–2.85)	1.099 (0.57–2.35)	0.9515 (0.56–2.49)	*p* < 0.005
Recipient				
Age	54 (2–85)	56 (8–81)	53 (1–86)	*p* = 0.002
Sex				*p* = 0.755
Male	3254 (60%)	1457 (62.2%)	9811 (61.9%)	
Female	2172 (40%)	884 (37.8%)	6038 (38.1%)	
HLA mismatches	3 (0–6)	3 (0–6)	5 (0–6)	*p* < 0.005
Delayed graft function				*p* < 0.005
No	3592 (66.2%)	1107 (47.3%)	9476 (59.8%)	
Yes	1834 (33.8%)	1234 (52.7%)	6373 (40.2%)	
Death -censored graft survival rate after 5 years	90.3%	90.6%	92.2%	*p* = 0.273
eGFR 3 Months, ml/min*173^2^	43.7 (1.4–340.7)	41.5 (1.4–279.2)	***	
eGFR 12 Months, ml/min*173^2^	46.1 (2.6–376.3)	45.2 (2.6–232.7)	***	
Number of Rejections			Rejection 1 year post-transpl.	
0	4830 (89%)	2069 (88.4%)	14911 (94.1%)	
1 or more	596 (11%)	272 (11.6%)	938 (5.9%)	
Previous kidney transplantation				
No	4578 (84.8%)	2040 (87.1%)	14102 (89%)	*p* = 0.009
Yes	848 (15.2%)	301 (12.9%)	1747 (11%)	

*Both lungs are counted as an individual organ.

**Value not reliable due to high number of missing values.

***Value not available.

Showing median + range or number + percentage.

UNOS database only contain DCD donors.

BMI, body mass index; CVA, cerebrovascular accident; GFR, glomerular filtration rate; KDRI, kidney donor risk index.

Extraction time was not available in a large percentage of the UNOS patients, this lead to a large exclusion in patients (215.987). The difference between the whole cohort and the selection we used is presented in Supplementary Table S1. There seems to be an overall similarity in donor characteristics such as age, sex, diabetes and hypertension. Striking is the difference seen in the number of extracted organs. In the data available for our analysis, fewer organs were procured for each donor than what is reported in the complete data. This might account for the difference seen between the Dutch NOTR database and the UNOS database with regard to extraction time, were UNOS had a shorter extraction time than the NOTR database (58 vs. 38 min). The NOTR had more comparable data on the number of extracted organs compared to the UNOS complete dataset, due to more complete registration of extraction times. More organs extracted per donor leads of course to an increase in average extraction time.

Median recipient age was similar in both datasets [NOTR: 54 (2–85) vs. UNOS: 53 (1–86), *p* = 0.058]. The number of HLA mismatches was significantly higher in the UNOS database (*p* < 0.005). There was also a significantly higher rate of delayed graft function in the recipients of DCD donor kidneys in the NOTR database compared to UNOS (NOTR: 52.7% vs. UNOS: 40.2%, *p* < 0.005). Overall graft survival did not differ between the DCD donors and all donors of the two cohorts though (5 years graft survival DCD only NOTR: 90.6% vs. UNOS 92.2%, *p* = 0.273 and NOTR: 90.3% vs. UNOS 92.2%, *p* = 0.151).

### NOTR Extraction Time

The impact of extraction time in the NOTR data, grouped per 10 min, on patient survival, graft survival and DGF is shown in Table 2. Increasing extraction times were significantly associated with a higher hazard rate of graft failure [HR 1.027 (1.004–1.050) *p* = 0.022] and odds for the development of DGF [OR 1.043 (1.021–1.066) *p* < 0.005]. These associations remained unchanged when adjusted for potential confounders (Table 2, models 1–5). Increasing extraction times were not significantly associated with a higher hazard rate of recipient death [HR 0.999 (0.981–1.0167) *p* = 0.916]. Interaction analysis on model 5 of Table 2 showed that the relationship between extraction time and the outcomes graft survival and DGF was not different for DBD and DCD (*p* = 0.111 and *p* = 0.080 respectively). Prolonged extraction times were associated with significantly lower eGFR values at both 3 months [B −0.305 (−0.519 to −0.092) *p* = 0.005] and 1 year [B −0.334 (−0.542 to −0.126) *p* = 0.002] post-transplantation in fully adjusted models (Supplementary Table S3). Analysis of eGFR values at 1 year is conducted with 432 missing cases, longer follow up with eGFR was not conducted because of too much missing cases. Increasing extraction times were not significantly associated with rejection post-transplantation. Next, multivariable analysis using model 4 of Table 2 was performed to examine the influence of prolonged extraction times on specific deceased donor-subgroups DBD and DCD (Supplementary Table S4). Increasing extraction times were not associated with a higher hazard rate of graft failure when the DBD and DCD groups were analyzed separately. A higher odds of developing DGF with increasing extraction time was only seen in the DCD group [OR 1.058 (1.030–1.087) *p* < 0.005].

**TABLE 2:** Multivariable Cox regression/binary logistic regression on extraction time (10 min) and patient survival, graft failure and DGF NOTR (DBD and DCD). Coefficients of full models are listed in Supplementary Table S1.

	Patient death HR [95% CI]	*p*	Graft failure HR [95% CI]	*p*	DGF	*p*
					OR [95% CI]	
Univariable	0.977 [0.960–0.994]	0.010	1.011 [0.989–1.034]	0.312	1.055 [1.036–1.075]	<0.005
Model 1	1.000 [0.982–1.018]	0.982	1.033 [1.011–1.056]	0.004	1.067 [1.047–1.088]	<0.005
Model 2	1.001 [0.983–1.019]	0.926	1.036 [1.016–1.059]	0.002	1.068 [1.048–1.089]	<0.005
Model 3	0.998 [0.980–1.016]	0.834	1.028 [1.006–1.051]	0.014	1.029 [1.008–1.051]	0.007
Model 4	1.000 [0.982–1.017]	0.956	1.028 [1.005–1.051]	0.016	1.030 [1.009–1.052]	0.006
Model 5	0.999 [0.981–1.017]	0.916	1.027 [1.004–1.050]	0.022	1.043 [1.021–1.066]	<0.005

Model 1: extraction time + donor age, BMI and gender.

Model 2: model 1 + cause of death*, donor diabetes, hypertension and last serum creatinine.

Model 3: model 2 + cold ischemic time, warm ischemic time, anastomosis time and number of reported organs**.

Model 4: model 3 + number of previous transplants, HLA mismatches, age recipient, gender recipient.

Model 5: model 4 + DBD/DCD.

*CVA, Trauma or other.

**Divided as <=2 or >2 organs.

BMI, body mass index.

### UNOS Extraction Time

Impact of extraction time in the UNOS data, grouped per 10 min, on patient survival, graft survival and DGF is shown in Table 3. Similarly to the Dutch database, UNOS data showed a significant association of prolonged extraction times with DGF [OR 1.036 (1.016–1.055) *p* < 0.005]. Prolonged extraction times were, however, not associated with a higher hazard rate of graft failure [HR 0.997 (0.970–1.025) *p* = 0.829] or a higher hazard rate of patient death in the UNOS data [HR 0.995 (0.971–1.019) *p* = 0.667]. Increasing extraction times were not significantly associated with acute rejection (*p* = 0.448) and rejection 1 year post-transplantation (*p* = 0.158).

**TABLE 3:** Multivariable Cox regression/binary logistic regression on extraction time (10 min) and patient survival, graft failure and DGF UNOS (DCD only). Coefficients of full models are listed in Supplementary Table S4.

	Patient death HR [95% CI]	*p*	Graft failure HR [95% CI]	*p*	DGF	*p*
					OR [95% CI]	
Univariable	0.967 [0.945–0.990]	0.004	0.981 [0.956–1.007]	0.156	1.004 [0.987–1.020]	0.663
Model 1	0.987 [0.964–1.010]	0.272	0.992 [0.967–1.018]	0.557	1.018 [1.000–1.035]	0.044
Model 2	0.987 [0.964–1.010]	0.256	0.992 [0.967–1.018]	0.559	1.022 [1.005–1.040]	0.013
Model 3	0.988 [0.965–1.013]	0.350	1.001 [0.974–1.029]	0.943	1.036 [1.017–1.055]	<0.005
Model 4	0.995 [0.971–1.019]	0.667	0.997 [0.970–1.025]	0.829	1.036 [1.016–1.055]	<0.005

Model 1: extraction time + donor age, BMI, ethnicity* and gender.

Model 2: model 1 + cause of death**, donor diabetes, hypertension and last serum creatinine.

Model 3: model 2 + cold ischemic time and number of recovered organs***.

Model 4: model 3 + previous transplants, HLA mismatches, recipient age and gender.

*African American or other.

**CVA, head trauma, anoxia or other.

***Divided as <=2 or >2 organs.

BMI, body mass index.

### NOTR Cut-off Value

**FIGURE 2:**
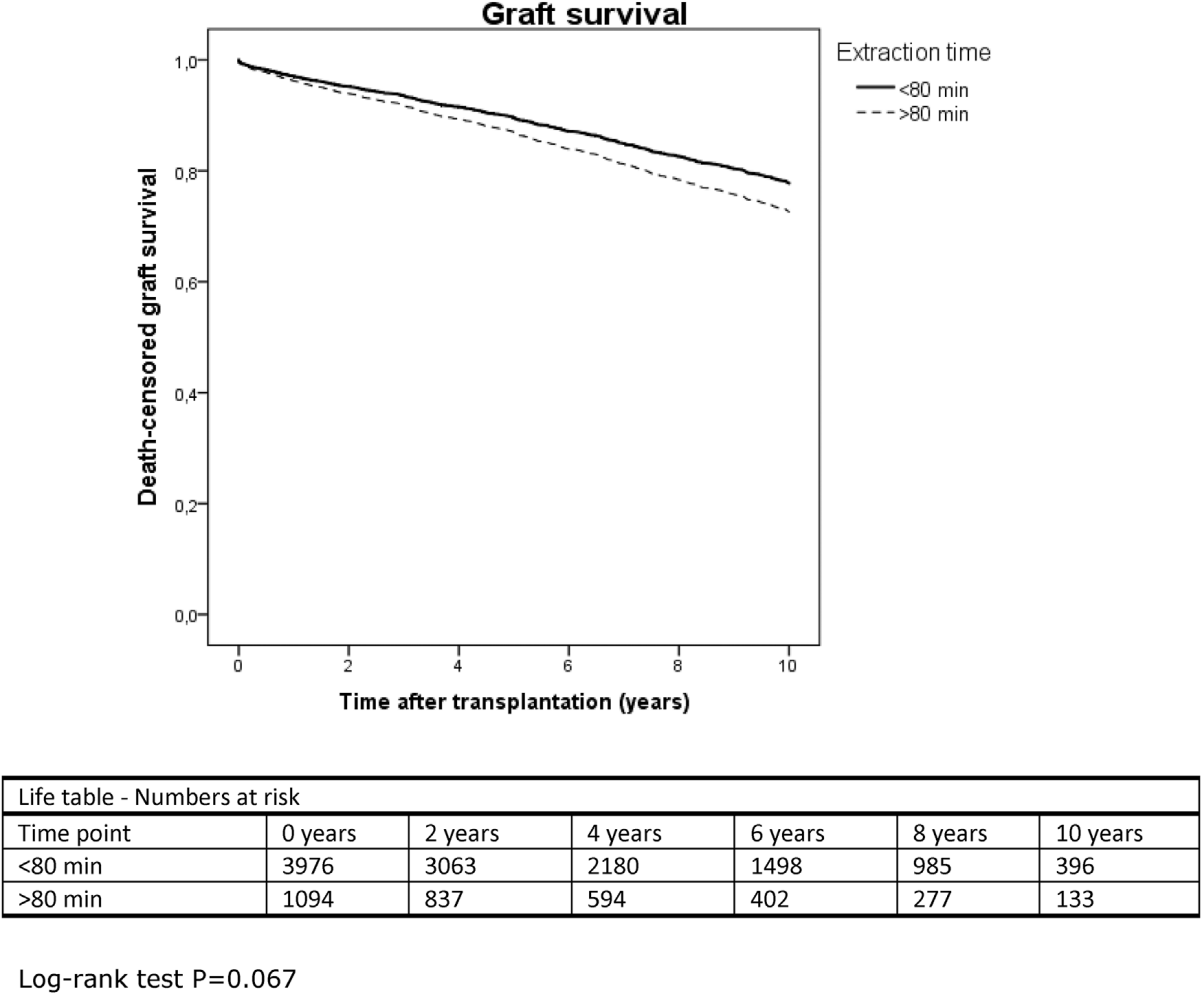
Kaplan-Meier curve displaying 10-years death-censored graft survival in the NOTR database, extraction time divided into over and under 80 min.

Multivariable Cox regression or binary logistic regression was used to find a cut-off value for the extraction time upper limit in the NOTR data. Extraction time was dichotomized divided into different time intervals between 40 and 110 min (Table 4) and analyses were performed using model 5 of Table 2, including all potential confounders. An extraction time over 80 min was associated with a 27.4% higher hazard rate of graft failure (8.0–50.2%; *p* = 0.004) (Figure 2); kidneys with an extraction time over 70 min had 23.7% higher odds of developing DGF (7.9–41.7%; *p* = 0.002), and those over 80 min as much as 43.8% higher odds (23.6–67.3%; *p* < 0.005).

**TABLE 4:** Multivariable Cox regression/binary logistic regression on binary extraction times and patient survival, graft failure and DGF NOTR (DBD and DCD).

Cut-off value (min)	% Patients per time interval (n = 5426)	Patient survival HR [95% CI]	*p*	Graft failure HR [95% CI]	*p*	DGF	*p*
						OR [95% CI]	
40	<40: 1016 (18.7%)	1.072 [0.940–1.222]	0.298	1.140 [0.957–1.370]	0.140	1.090 [0.929–1.279]	0.291
	>40: 4410 (81.3%)						
50	<50: 1953 (36%)	1.040 [0.932–1.160]	0.482	1.013 [0.877–1.171]	0.856	1.013 [0.877–1.171]	0.856
	>50: 3473 (64%)						
60	<60: 2928 (54%)	1.013 [0.911–1.128]	0.807	1.061 [0.921–1.222]	0.410	1.110 [0.976–1.261]	0.111
	>60: 2498 (46%)						
70	<70: 3708 (68.3%)	0.898 [0.883–1.115]	0.898	1.126 [0.969–1.308]	0.122	1.237 [1.079–1.417]	0.002
	>70: 1718 (31.7%)						
80	<80: 4246 (78.3%)	1.050 [0.920–1.199]	0.470	1.274 [1.080–1.502]	0.004	1.438 [1.236–1.673]	<0.005
	>80: 1180 (21.7%)						
90	<90: 4617 (85.1%)	1.039 [0.890–1.212]	0.630	1.258 [1.038–1.523]	0.019	1.428 [1.199–1.700]	<0.005
	>90: 809 (14.9%)						
100	<100: 4833 (89%)	0.974 [0.813–1.165]	0.771	1.239 [0.995–1.543]	0.055	1.337 [1.095–1.631]	0.004
	>100: 593 (11%)						
110	<110: 4996 (92%)	0.912 [0.738–1.128]	0.395	1.292 [1.009–1.654]	0.042	1.501 [1.194–1.887]	<0.005
	>110: 430 (8%)						

Model: extraction time + donor age, BMI, gender, cause of death, diabetes, hypertension and last serum creatinine, cold ischemic time, warm ischemic time, anastomosis time, number of reported organs, number of previous transplants, HLA mismatches; recipient age, gender and DBD/DCD.

BMI, body mass index.

### UNOS Cut-off Value

A similar analysis was performed on the UNOS database to find a cut-off value for extraction time, using model 4 of Table 3 (Table 5). An extraction time over 30 min was associated with 14.5% higher odds of developing DGF (1.063–1.233; *p* < 0.005).

**TABLE 5:** Multivariable Cox regression/binary logistic regression on binary extraction times and patient survival, graft failure and DGF UNOS (DCD only).

Cut-off value (min)	% Patients per time interval (n = 15849)	Patient survival HR [95% CI]	*p*	Graft failure HR [95% CI]	*p*	DGF	*p*
						OR [95% CI]	
20	<20: 1189 (7.5%)	0.906 [0.773–1.061]	0.220	1.043 [0.861–1.264]	0.667	1.056 [0.931–1.197]	0.396
	>20: 14660 (92.5%)						
30	<30: 4823 (30.4%)	0.999 [0.905–1.103]	0.988	1.008 [0.900–1.129]	0.890	1.145 [1.063–1.233]	<0.005
	>30: 11026 (69.6%)						
40	<40: 8871 (56%)	1.033 [0.940–1.135]	0.501	0.995 [0.894–1.108]	0.928	1.182 [1.102–1.268]	<0.005
	>40: 6978 (44%)						
50	<50: 11812 (74.5%)	0.932 [0.835–1.041]	0.215	0.941 [0.831–1.067]	0.344	1.116 [1.031–1.208]	0.007
	>50: 4037 (25.5%)						
60	<60: 13588 (85.7%)	1.021 [0.892–1.168]	0.768	1.024 [0.882–1.188]	0.755	1.146 [1.040–1.263]	0.006
	>60: 2261 (14.3%)						
70	<70: 14691 (92.7%)	0.934 [0.780–1.119]	0.461	0.953 [0.783–1.160]	0.630	1.061 [0.933–1.207]	0.369
	>70: 1158 (7.3%)						
80	<80: 15214 (96%)	0.884 [0.697–1.121]	0.310	0.913 [0.706–1.180]	0.486	1.138 [0.961–1.347]	0.134
	>80: 635 (4%)						
90	<90: 15533 (98%)	0.865 [0.621–1.206]	0.393	0.719 [0.487–1.062]	0.098	1.133 [0.895–1.434]	0.298
	>90: 316 (2%)						

Model: extraction time + donor age, BMI, ethnicity, gender, cause of death, diabetes, hypertension, last serum creatinine, cold ischemic time, number of recovered organs, previous transplants, HLA mismatches; recipient age and gender.

BMI, body mass index.

### Factors Influencing Extraction Time

Multivariable linear regression was performed to explore factors that significantly influenced extraction time. The analysis was performed in a merged dataset, which combined data of all kidney transplant donors and recipients of both the NOTR and UNOS databases. The variables donor gender, BMI, history of hypertension and diabetes and NOTR vs. UNOS database (DCD only) were used in the regression analysis. In this combined dataset, all the aforementioned factors together accounted for 172% of the variability in extraction time [R^2^ = 0.172, adjusted R^2^ = 0.1172, F (5, 21235) = 880.5, *p* < 0.005]. Nonstandardized (B) and standardized (β) regression coefficients for each predictor in the regression model are reported in Supplementary Table S6. The largest contributor to a longer extraction time was country of the donation, NOTR (the Netherlands) vs. UNOS (United States) [B −23.557 (−24.274, −22.841) *p* < 0.005].

## Discussion

Prolonged postmortem kidney extraction times in the Dutch NOTR database, increasing per 10 min, were associated with a higher hazard rate of graft loss, delayed graft function and lower eGFR values 3 months and 1 year after transplantation. Extraction times over 80 min in DBD and DCD donors combined, significantly increased the hazard rate of graft loss and the odds of developing DGF compared to extraction times lower than 80 min. A large difference in the median extraction time was seen between the Dutch donors and donors from America (58 vs. 38 min). In the UNOS database, increasing extraction time in DCD donors was not associated with patient survival and graft survival but increased the odds of developing DGF. In addition, extraction times over 30 min showed increased odds of developing DGF compared to extraction times under 30 min.

Our data suggest that, in addition to other factors, extended kidney extraction time is an important variable that determines deceased donors’ kidney transplantation outcome. It was not possible to state a universal extraction time cut-off value for all kidney donors. It nonetheless seems that extraction times higher than 80 min lead to a greater odds of developing DGF and a higher hazard of developing graft failure in Dutch transplant recipients. This means that postmortem donor operation times should be kept as short as possible in similar cases. In addition, preservation during this time-period could be improved, especially in prolonged extraction times. When analyzing the data of the cut-off value, we need to take in consideration that there might be a loss of impact at the higher and lower extraction times in the UNOS database (e.g., <20 min and >70 min), where just over 7% of the patients are in one group and the rest are in the other. This could explain why the association between extraction time and DGF is lost >70 min in the UNOS database.

The different effect of prolonged extraction time on outcomes in both databases is likely caused by the inequality between the two organ donation and transplantation systems (Table 1). A factor that influences transplantation outcome adversely, i.e., increased donor age, (14) was higher in the NOTR database, while cold ischemic time (15–17) was longer in the UNOS database. KDRI was calculated for a better understanding of the differences in kidney donor quality resulting from different baseline characteristics. KDRI combines ten donor factors and gives a validated estimate of the relative risk of post-transplantation kidney graft failure (12, 13). NOTR donors had a significantly higher KDRI_median_ value than UNOS donors, indicating a higher relative risk of post-transplantation kidney graft failure and suggesting an average inferior quality of transplanted kidneys in the Dutch NOTR database. The difference in KDRI value is a plausible explanation for the different influence of prolonged extraction time on transplantation outcome between the two groups, where increased extraction time could have a detrimental effect on transplantation outcome if the donor kidney was already more susceptible to graft failure.

Besides the difference in KDRI value, extraction times too are different between the databases, with a median value of 58 min for NOTR compared to 38 min for UNOS. A prolonged extraction time could be the result of more organs being procured from each individual donor in the Dutch cohort compared to the American cohort, or might be explained by differences in donation procedures. Since NOTR shows reported number of organs and UNOS extracted number of organs, a comparison between the two databases may not be entirely correct. The lower number of extracted donor organs in the UNOS data compared to the number of reported organs in the NOTR data could be explained by the fact that not all reported organs are always procured, due to some degree of organ discard prior to retrieval. There is no clear explanation as to why extraction times differed, the experience of surgeons was not measured, and the specific surgical procedure was not part of our analysis. Given that, when looking at kidney-only procurement, the median time of kidney extraction was still longer in the NOTR database (52 vs. 33 min) while possible operating time-increasing variables (such as male gender and BMI) (18, 19) were less favorable among US donors, a relevant difference in expertise and/or surgical technique cannot be ruled out. In addition, by only using a selection of the full UNOS database due to limited available extraction times, selection bias might be introduced. There is a difference in number of extracted organs between the full UNOS database and the cases we used for our calculation. The more organs extracted, the longer the extraction time, so we might underreported the actual extraction time for the full UNOS database and thereby the possible effect of extraction time on transplantation outcome. This might also explain the difference seen in cut-off point between the NOTR and UNOS (80 vs. 30 min).

To the best of our knowledge, only a few other studies have focused on the effect of extraction time on kidney transplantation outcome. A previous study, conducted on smaller patient cohort (n = 576), emphasized the influence of extraction times higher than 60 min on the occurrence of DGF (8). Another study by Heylen et al., found that prolonged extraction time was associated with graft loss after donation after circulatory death (DCD), but not after brain death donation (DBD) (10). This analysis was performed on the Eurotransplant region which includes the Netherlands, between 2004 and 2013. Although it is performed in an overlapping time interval, Heylen et al. does not show an association between extraction time and graft loss in DBD and DCD donors combined as we do. In the NOTR database, prolonged extraction time lost its association when the database was split into DBD or DCD donors only. The association between prolonged extraction times and the occurrence of DGF remained only in the DCD group. When analyzing the whole NOTR database, with DBD/DCD as a covariate in the multivariable analysis, the association of kidney extraction time with both graft failure and DGF remained significant. This could mean that by dividing the NOTR database into two groups the number of donors became too small to maintain enough power for the graft failure analysis. Interaction analyses showed that the relationship between extraction time and graft survival and DGF was not different for DBD and DCD. Although, by performing statistical analysis on a combined group we cannot rule out that we have measured an artificial effect of extraction time on transplantation outcome, even though we corrected for the donor type in our analysis. Apart from a slightly different outcome in graft survival between the study by Heylen et al. and ours, we were able to perform additional analyses on the outcome DGF, patient survival and eGFR, giving more insight on the impact of extraction time on kidney transplantation outcome.

Besides kidney extraction time, hepatectomy time has also been associated with impaired transplantation outcome (7). Donor risk index was used by Jochmans et al. as a marker for organ quality, showing that livers from DCD and higher-risk donors are most affected by prolonged extraction time (7). This is in line with the results from Heylen on nephrectomy time and our obtained data in the NOTR database, where the KDRI was higher than in the UNOS database and the effect of prolonged extraction times on transplantation outcome was stronger.

More research needs to be conducted on how to improve or at least maintain organ quality during the period of extraction. Flushing the organ via the aorta in a fairly warm body results in sub-normothermic conditions which are most likely suboptimal for organ preservation. Higher organ temperatures result in higher metabolism, (3) and in current organ retrieval practice the kidneys receive no oxygen or nutrients, which causes a discrepancy between cellular demand and supply. Better temperature control during the extraction or otherwise reducing kidney metabolism, for example with the use of hydrogen sulphide, (20) could improve transplantation outcome even with longer extraction times. Since shortening of organ extraction time may not always be feasible, future research should focus on alternative improvements that protect the kidneys during organ procurement.

After an analysis among Dutch donors, the hepatectomy time proved to be a significant independent risk factor for the development of non-anastomotic biliary strictures after DCD liver transplantation (21). This led to the implementation of a new protocol, combined with extra training of surgeons and creating awareness on this important and potentially modifiable risk factor. By creating awareness that extraction time is an important factor that could influence transplantation outcome, extraction times themselves could be reduced.

A limitation of this study is the nature of its design. The large cohort size ensures a good power to find significant associations, but does not establish causality. We show different results in the different cohorts, therefore the results should be interpreted with care since generalization is not possible. In addition, not all data were fully available in the two databases, and UNOS only had data on extraction times of DCD donors. The large number of extraction times that were not available in the UNOS database could have induced bias regarding this analysis. Also, several other subtle differences existed in how data were stored in the databases—in some cases, data values were missing and data had to be imputed. This could have introduced bias, although in our opinion not all differences between the databases can be explained by these dissimilarities. Also, there could be an immeasurable bias in the prolonged extraction time of donors itself. Factors that predispose the fact that they needed a longer extraction time could explain the generally worse transplantation outcome instead of the prolonged extraction time itself. Even so, extraction time is an easily measured variable that a transplantation professional can take into account in the decision to accept a donor kidney or not. If these unmeasured factors contribute to a worse transplantation outcome but also to a prolonged extraction time, extraction time itself is still a variable to contemplate and should be taken into account.

In conclusion, extraction time during deceased donor procedures was associated with graft loss, delayed graft function and lower eGFR values in Dutch kidney transplant recipients, and with delayed graft function in American transplant recipients. Prolonged extraction time seems a potentially important determinant of kidney transplantation outcome, especially in kidneys recovered from high-risk donors.

## Capsule Summary Sentence

The aim of the present manuscript was to investigate the impact of kidney extraction time on eGFR, delayed graft function (DGF), graft failure, and patient survival after renal transplantation. We analyzed this in two large cohorts of both Netherlands (5,426 transplant procedures) and United States (15,849 transplant procedures). Our results show that prolonged extraction time increases the risk of DGF in both Dutch and American recipients and even leads to an elevated graft failure rate in Dutch recipients. In addition, longer extraction times were associated with lower eGFR values after transplantation. We believe that our manuscript demonstrates the detrimental influence of a potentially modifiable surgical factor during deceased donor organ donation. Shortening kidney extraction times could improve renal transplantation outcome.

## Data Availability

The data analyzed in this study is subject to the following licenses/restrictions: The data that support the findings of this study are available on request from the UNOS and/or NOTR. Requests to access these datasets should be directed to info@transplantatiestichting.nl and UNOS.org.

## Abbreviations

BMI, Body mass index; DBD, Donation after brain death; DCD, Donation after circulatory death; DGF, Delayed graft function; eGFR, Estimated glomerular filtration rate; HCV, Hepatitis C virus; KDRI, Kidney donor risk index; NOTR, Dutch Organ Transplant Registry; UNOS, United Network for Organ Sharing.
