# Supplementary material for: Prolonged Organ Extraction Time Negatively Impacts Kidney Transplantation Outcome
Source: Transpl Int. 2022 Feb 9;35:10186. doi: 10.3389/ti.2021.10186 (PMC8863594; doi:10.3389/ti.2021.10186)
Supplement: Supplementary file 1 [file DataSheet1.docx]

| **Supplementary Table 1. Donor Characteristics of UNOS all cases and UNOS used cases** | | | |
| --- | --- | --- | --- |
| **Characteristics** | **UNOS all cases (215987)** | **UNOS used cases (15849)** |  |
| **DONOR** |  |  |  |
| Age, yrs | 39 (0-93) | 39 (1-69) |  |
| Sex |  |  |  |
| Male | 129083 (59.8%) | 10542 (66.5%) |  |
| Female | 86904 (40.2%) | 5307 (33.5%) |  |
| BMI | 25.6 (7.16-74.36) | 26.9 (8.91-69.2) |  |
| Donor Type |  |  |  |
| DBD | 168687 (78.1%) |  |  |
| DCD | 18783 (8.7%) | 15849 (100%) |  |
| Cause of Death |  |  |  |
| CVA | 79023 (36.6%) | 2455 (15.5%) |  |
| Trauma |  |  |  |
| Head trauma | 75799 (35,1%) | 5089 (32.1%) |  |
| Anoxia | 46663 (21.6%) | 7543 (47.6%) |  |
| Other | 14381 (6.7%) | 762 (4.8%) |  |
| Hypertension |  |  |  |
| Yes | 57938 (26.8%) | 3778 (23.8%) |  |
| No | 127785 (59.2%) | 12071 (76.2%) |  |
| Diabetes |  |  |  |
| Yes | 18001 (9,6%) | 903 (5.7%) |  |
| No | 168166 (77.9%) | 14946 (94.3%) |  |
| Extracted number of organs |  |  |  |
| 1 | 15283 (7.1%) | 56 (0.4%) |  |
| 2 | 33337 (15.4%) | 8657 (54.6%) |  |
| 3 | 74875 (34.7%) | 5672 (35.8%) |  |
| 4 | 41764 (19.3%) | 866 (5.5%) |  |
| 5 | 25786 (11.9%) | 527 (3.3%) |  |
| 6 | 14019 (6.5%) | 71 (0.4%) |  |
| 7 | 10420 (4.8%) |  |  |
| Warm ischemic time (DCD only), min | 15 (0-180)* | 17 (0-180)* |  |
| Cold ischemic time, min | 780 (0-11220) | 1080 (0.6-5940) |  |
| Extraction time, min | N.A. | 38 (5-259) |  |
| Extraction time kidney-only donation, min | N.A. | 33 (6-165) |  |
| KDRI_median_ | 0.9923 (0.41-4.27) | 0.9515 (0.56-2.49) |  |
| Showing median + range or number + percentage. UNOS database only contain DCD donors.  BMI = body mass index.  CVA = cerebrovascular accident.  GFR = glomerular filtration rate.  KDRI = kidney donor risk index. *value not reliable due to high number of missing values. | | |  |

| **Supplementary Table 2. Multivariable Cox regression/binary logistic regression on extraction time (10 min) and patient survival, graft failure and DGF NOTR (DBD and DCD)** | | | | | | |
| --- | --- | --- | --- | --- | --- | --- |
|  | Patient death  HR [95% CI] | P | Graft failure  HR [95% CI] | P | DGF  OR [95% CI] | P |
| Extraction time | 0.999 [0.981-1.017] | 0.916 | 1.027 [1.004-1.050] | 0.022 | 1.043 [1.021-1.066] | <0.005 |
| Donor age | 1.010 [1.006-1.015] | <0.005 | 1.023 [1.017-1.029] | <0.005 | 1.017 [1.012-1.022] | <0.005 |
| Donor gender | 1.124 [1.008-1.253] | 0.036 | 1.094 [0.949-1.260] | 0.261 | 0.902 [0.791-1.030] | 0.127 |
| Donor BMI | 1.002 [0.989-1.016] | 0.757 | 1.015 [0.998-1.032] | 0.077 | 1.017 [1.001-1.033] | 0.034 |
| Donor hypertension | 1.047 [0.923-1.188] | 0.474 | 1.132 [0.960-1.336] | 0.141 | 1.279 [1.095-1.493] | 0.002 |
| Last serum creatinine donor | 1.002 [1.001-1.003] | 0.004 | 1.002 [1.001-1.003] | 0.001 | 1.006 [1.004-1.007] | <0.005 |
| Cause of death* | 1.069 [0.998-1.145] | 0.058 | 1.054 [0.963-1.155] | 0.253 | 1.010 [0.933-1.095] | 0.800 |
| Donor diabetes | 1.026 [0.904-1.164] | 0.691 | 1.126 [0.963-1.316] | 0.137 | 0.978 [0.824-1.161] | 0.797 |
| Cold ischemic time | 1.000 [1.000-1.000] | 0.325 | 1.000 [1.000-1.001] | 0.001 | 1.001 [1.000-1.001] | <0.005 |
| Warm ischemic time | 1.004 [0.992-1.016] | 0.553 | 1.011 [0.995-1.026] | 0.172 | 1.030 [1.015-1.045] | <0.005 |
| Anastomosis time | 1.008 [1.005-1.012] | <0.005 | 1.005 [1.000-1.010] | 0.049 | 1.009 [1.004-1.014] | 0.001 |
| Number of reported organs** | 1.088 [0.932-1.270] | 0.284 | 0.856 [0.700-1.048] | 0.132 | 0.891 [0.735-1.079] | 0.236 |
| Number of previous transplants | 1.208 [1.077-1.356] | 0.001 | 1.347 [1.194-1.521] | <0.005 | 1.385 [1.222-1.570] | <0.005 |
| HLA mismatches | 1.074 [1.034-1.114] | <0.005 | 1.106 [1.051-1.164] | <0.005 | 1.066 [1.018-1.117] | 0.007 |
| Recipient age | 1.059 [1.054-1.064] | <0.005 | 0.990 [0.985-0.995] | <0.005 | 0.999 [0.994-1.004] | 0.703 |
| Recipient gender | 0.882 [0.792-0.981] | 0.021 | 1.002 [0.872-1.151] | 0.981 | 0.875 [0.770-0.993] | 0.039 |
| DBD or DCD | 0.959 [0.747-1.231] | 0.741 | 0.915 [0.665-1.259] | 0.584 | 2.963 [2.219-3.959] | <0.005 |

*CVA, trauma or other

**Divided as <=2 or >2 organs

BMI = body mass index.

| **Supplementary Table 3. Multivariable linear regression on extraction time (10 min) and eGFR NOTR (DBD and DCD)** | | | | |
| --- | --- | --- | --- | --- |
|  | eGFR 3 months  B [95% CI] | P | eGFR 1 year  B [95% CI] | P |
| Univariable | 0.599 [0.339 to 0.859] | <0.005 | 0.666 [0.402 to 0.931] | <0.005 |
| Model 1 | -0.447 [-0.665 to -0.229] | <0.005 | -0.446 [-0.658 to -0.234] | <0.005 |
| Model 2 | -0.467 [-0.685 to -0.249] | <0.005 | -0.465 [-0.677 to -0.254] | <0.005 |
| Model 3 | -0.235 [-0.452 to -0.017] | 0.034 | -0.301 [-0.513 to -0.089] | 0.005 |
| Model 4 | -0.272 [-0.483 to -0.061] | 0.012 | -0.317 [-0.523 to -0.112] | 0.002 |
| Model 5 | -0.305 [-0.519 to -0.092] | 0.005 | -0.334 [-0.542 to -0.126] | 0.002 |

Model 1: extraction time + donor age, BMI and gender

Model 2: model 1 + cause of death*, donor diabetes, hypertension and last serum creatinine

Model 3: model 2 + cold ischemic time, warm ischemic time, anastomosis time and number of reported organs**

Model 4: model 3 + number of previous transplants, HLA mismatches, recipient age and gender

Model 5: model 4 + DBD/DCD

*CVA, trauma or other

**Divided as <=2 or >2 organs

BMI = body mass index.

| **Supplementary Table 4. Multivariable Cox regression/binary logistic regression on extraction time (10 min) and patient survival, graft failure and DGF NOTR (DBD and DCD)** | | | | | | |
| --- | --- | --- | --- | --- | --- | --- |
|  | Patient death  HR [95% CI] | P | Graft failure  HR [95% CI] | P | DGF  OR [95% CI] | P |
| DBD | 1.024 [0.996-1.052] | 0.090 | 1.030 [0.993-1.068] | 0.118 | 1.027 [0.990-1.066] | 0.149 |
| DCD | 0.980 [0.957-1.005] | 0.114 | 1.024 [0.994-1.055] | 0.121 | 1.058 [1.030-1.087] | <0.005 |

Model: extraction time + donor age, BMI, gender, cause of death*, diabetes, hypertension, last serum creatinine, cold ischemic time, first and second warm ischemic time and number of reported organs**, number of previous transplants, HLA mismatches, recipient age and gender. BMI = body mass index.

*CVA, trauma or other

**Divided as <=2 or >2 organs

| **Supplementary Table 5. Multivariable Cox regression/binary logistic regression on extraction time (10 min) and patient survival, graft failure and DGF UNOS (DCD only)** | | | | | | |
| --- | --- | --- | --- | --- | --- | --- |
|  | Patient death  HR [95% CI] | P | Graft failure  HR [95% CI] | P | DGF  OR [95% CI] | P |
| Extraction time | 0.995 [0.971-1.019] | 0.667 | 0.997 [0.970-1.025] | 0.829 | 1.036 [1.018-1.055] | <0.005 |
| Donor age | 1.012 [1.009-1.016] | <0.005 | 1.012 [1.008-1.017] | <0.005 | 1.017 [1.014-1.020] | <0.005 |
| Donor gender | 0.981 [0.890-1.080] | 0.690 | 1.066 [0.957-1.188] | 0.246 | 0.795 [0.740-0.854] | <0.005 |
| Donor BMI | 0.999 [0.992-1.006] | 0.846 | 1.002 [0.994-1.009] | 0.692 | 1.021 [1.016-1.026] | <0.005 |
| Ethnicity* | 1.526 [1.294-1.801] | <0.005 | 1.438 [1.203-1.720] | <0.005 | 0.955 [0.838-1.089] | 0.495 |
| Cause of death** | 1.026 [0.980-1.074] | 0.277 | 1.030 [0.979-1.085] | 0.254 | 1.004 [0.971-1.038] | 0.812 |
| Donor diabetes | 0.974 [0.807-1.175] | 0.780 | 1.402 [1.164-1.688] | <0.005 | 1.032 [0.894-1.190] | 0.669 |
| Donor hypertension | 1.049 [0.940-1.171] | 0.392 | 1.362 [1.202-1.542] | <0.005 | 1.156 [1.064-1.257] | 0.001 |
| Last serum creatinine donor | 0.999 [0.998-1.000] | 0.078 | 1.000 [0.999-1.001] | 0.803 | 1.003 [1.002-1.003] | <0.005 |
| Cold ischemic time | 1.000 [1.000-1.000] | 0.002 | 1.000 [1.000-1.000] | <0.005 | 1.000 [1.000-1.000] | <0.005 |
| Number of recovered organs*** | 1.011 [0.915-1.117] | 0.832 | 0.910 [0.812-1.020] | 0.106 | 0.902 [0.839-0.971] | 0.006 |
| Number of previous transplants | 0.769 [0.663-0.892] | 0.001 | 0.913 [0.780-1.069] | 0.256 | 0.759 [0.683-0.844] | <0.005 |
| HLA mismatches | 0.979 [0.948-1.011] | 0.202 | 1.080 [1.038-1.123] | <0.005 | 1.091 [1.065-1.118] | <0.005 |
| Recipient age | 1.048 [1.044-1.053] | <0.005 | 0.981 [0.977-0.985] | <0.005 | 1.000 [0.997-1.003] | 0.945 |
| Recipient gender | 0.859 [0.782-0.943] | 0.001 | 0.920 [0.828-1.022] | 0.119 | 0.664 [0.620-0.711] | <0.005 |

*African American or other

**CVA, head trauma, anoxia or other

***Divided as <=2 or >2 organs

BMI = body mass index.

| **Supplementary Table 6. Multivariable linear regression on extraction time and potential influencing variables.** | | | |
| --- | --- | --- | --- |
| Variable | B [95% CI] | β | P |
| Men vs. women | -3.413 [-4.045, -2.780] | -0.067 | <0.005 |
| Donor BMI | 0.002 [-0.047, 0.050] | 0.000 | 0.945 |
| NOTR (DCD) vs. UNOS (DCD) | -23.557 [-24.274, -22.841] | -0.415 | <0.005 |
| Donor hypertension | -2.159 [-2.905, -1.412] | -0.037 | <0.005 |
| Donor diabetes | -2.370 [-3.502, -1.237] | -0.026 | <0.005 |
